# Clinical Aniseikonia in Anisometropia and Amblyopia

**DOI:** 10.22599/bioj.154

**Published:** 2020-11-20

**Authors:** Jayshree South, Tina Gao, Andrew Collins, Arier Lee, Jason Turuwhenua, Joanna Black

**Affiliations:** 1 The University of Auckland, NZ

**Keywords:** Aniseikonia, Anisometropia, Amblyopia, Binocular Vision, Suppression

## Abstract

**Purpose:**

Clinically, aniseikonia (a perceived difference in shape and image size between the eyes) is often neglected in anisometropic amblyopia due to assumed measurement difficulties. Therefore, we currently lack evidence on whether correction of aniseikonia is beneficial. This study aimed to determine whether subjective aniseikonia is measurable in anisometropia with or without amblyopia.

**Methods:**

Participants (15–52 years) with Anisometropic Amblyopia (n = 7), Anisometropia without amblyopia (n = 6) and Isometropic Controls (n = 6) were recruited. Subjective aniseikonia was measured using three clinical techniques: Robertson Technique (RT) (penlight and Maddox rod), Aniseikonia Inspector Version 3 (AI3), and the New Aniseikonia Test booklet (NAT), and a psychophysical adaptive method, the Contrast-balanced Aniseikonia Test (CAT), where dichoptic contrast adjustments compensate for any suppression.

**Results:**

Eighteen participants completed all tests, one Anisometropic Amblyopia participant could only complete the CAT and NAT due to fusion loss. The Anisometropic Amblyopia group exhibited the most aniseikonia (range –1.50–+10.50%) followed by Anisometropic Controls (range –3.30–+4.50%) and Isometropic Controls (range –1.50–+3.28%). There was a significant trend of more subjective aniseikonia with increasing amounts of anisometropia across all four tests (AI3 r = 0.630, p = 0.005; NAT r = 0.542, p = 0.017; RT r = 0.499, p = 0.035; CAT r = 0.440, p = 0.059. Bland Altman analysis demonstrated clinically significant levels of variability between the tests.

**Conclusions:**

Subjective aniseikonia can be reliably measured in patients with anisometropia and suppression. Subjective aniseikonia measurement is recommended as four of the most commonly used clinical tests did not support the 1% per dioptre rule of thumb.

## Introduction

Aniseikonia is a binocular vision disorder where images perceived by the two eyes differ in size and/or shape. In the context of anisometropia, aniseikonia can result from inherent anatomical differences (axial length and/or refractive components within the eye), differences in photoreceptor spacing between eyes, and cortical adaptations. Aniseikonia can also be optically induced by spectacles or contact lenses used in the correction of anisometropia. The patient’s perceived aniseikonia, as measured clinically or using psychophysical methods, is a product of all of these factors.

Anisometropia occurs when there is a significant difference in refractive error between the two eyes (defined as a difference of greater than 1.00DS in spherical equivalent) and increases the risk of amblyopia in young children (Caputo et al. 2007; Donahue 2005; Ingram RM 1979; Weakley 2001). The resulting unequal focus results in persistent blur on one retina, which leads to suppression. Anisometropia is refractive and/or axial in origin, and the type affects the theoretical sizes of the retinal images leading to perceived aniseikonia. The difference in image sizes hinders binocular vision and may further stimulate suppression. Chronic suppression due to blur and possibly unequal image size leads to the development of amblyopia.

Clinically, the first step in standard amblyopia treatment involves correction of refractive error (Cotter et al. 2006; Moseley et al. 2002; Royal College of Ophthalmologists March 2012; Stewart CE 2004; Wallace et al. 2018). Approximately 30% of children achieve equal visual acuity with refractive correction alone (Chen et al. 2007; Cotter et al. 2006; Cotter et al. 2012; Stewart CE 2004). The remaining 70% of children require additional occlusion or penalisation treatments. However, even though the current standard treatments can be effective, approximately half of treated children are left with residual deficits in visual acuity, and most do not achieve age-normal stereoacuity, despite good compliance with amblyopia treatment (Scheiman et al. 2005; Scheiman et al. 2008; Wallace et al. 2011). Given that about two-thirds of children with amblyopia have anisometropia, and children with anisometropia are likely to experience both anatomical and spectacle-induced aniseikonia, it is possible that aniseikonia may be a barrier to binocularity, stimulating suppression and limiting binocular visual improvement. Correcting aniseikonia along with anisometropia may improve visual outcomes, but this has not been directly investigated in children previously and is not considered in current clinical guidelines for amblyopia treatment.

Accurate measurement of aniseikonia is often not attempted in anisometropic amblyopes, as subjective aniseikonia tests are often thought to be too difficult to administer in these patient groups due to age, poor vision, or absent binocular vision (Davis 1959). Most aniseikonia measurement tools rely on direct comparison of images seen by each eye, requiring simultaneous binocular perception or stereopsis. It is assumed that direct comparison tasks are not possible in amblyopes due to the image from the amblyopic eye being too poor in quality or too strongly suppressed for the binocular image size difference to be recognised. As a result, few studies (Lubkin et al. 1999; Romano and Kohn 1972) have attempted to measure aniseikonia in non-fusing participants. Instead, clinicians often rely on estimations or empirical calculations of aniseikonia using the anisometropic difference in refractive error. The common clinical rule of thumb is 1% of aniseikonia per dioptre of spherical anisometropic difference (Berens and Bannon 1963; Ogle 1950). This rule is based solely on theoretical optics (Davis 1959; Ryan 1975), which overestimates aniseikonia and can be misleading. These estimations do not account for retinal differences (Benegas et al. 1999; Okamoto et al. 2014), cortical adaptations (Bradley et al. 1983), or any compounded aniseikonia induced by the spectacle corrections. Therefore, calculations from refractive error alone do not provide an accurate solution for the management of aniseikonia.

Children with anisometropia are rarely symptomatic of aniseikonia, which may be due to cortical adaptations such as suppression. However, strong suppression is more associated with strabismus or stimulus deprivation amblyopia. Whereas patients with anisometropic amblyopia and no strabismus often demonstrate lower levels of suppression, allowing for limited binocular functions such as fusion, gross stereopsis, and some reduced binocular summation (Donahue 2005; Levi et al. 2011; Weakley 2001).

Recent investigations into binocular treatment methods for unilateral amblyopia have demonstrated that binocular mechanisms are intact but are suppressed in order to cope with dissimilar images in the two eyes. The unbalanced interocular suppression associated with amblyopia can be overcome by adjusting the image contrast or luminance dichoptically presented to each eye, until the targets from either eye become simultaneously visible and equally salient (Harauzov et al. 2010; He et al. 2006; Hess et al. 2010; Mansouri et al. 2014; Maya-Vetencourt et al., 2012). Given that binocular mechanisms appear intact in anisometropic amblyopia, subjective aniseikonia should be measurable as long as suppression can be overcome during testing.

Internationally there does not appear to be a ‘gold standard’ test used for the measurement of aniseikonia. The Aniseikonia Inspector version 3 (AI3) (Optical Diagnostics, Culemborg, The Netherlands) (Kehler et al. 2014) and the New Aniseikonia Test (NAT) (Good-Lite Company, Tokyo, Japan) (McCormack et al. 1992) are two of the more routinely used clinical tests, but there is a lack of evidence around test comparisons and reliability between tests. In this study we investigated the use of 4 different subjective aniseikonia tests on three groups of participants: those with anisometropic amblyopia, anisometropia and no amblyopia, and isometropic controls. Our aim was to assess whether subjective aniseikonia can be successfully measured in anisometropic amblyopia and to examine the correlations between the four aniseikonia tests and refractive error.

## Methodology

This study was approved by the University of Auckland Human Participants Ethics Committee and adhered to the principles of the Declaration of Helsinki. Written informed consent was obtained for adult participants (16 years and over) and parents/guardians of child participants, and verbal assent was obtained for child participants.

### Participants

19 participants (age range 15–52 years) with healthy eyes and no previous history of eye surgery were recruited into 3 study groups: 1) Anisometropic amblyopia, 2) Anisometropia control, and 3) Isometropic control. Recruitment of participants was through the University of Auckland optometry clinic and local optometrist and orthoptic referrals.

### Group Criteria

- **Anisometropic Amblyopia group:** best corrected visual acuity (BCVA) in the amblyopic eye of ≥0.20 logMAR and the fellow eye ≤0.10 logMAR, and an interocular difference of ≥0.2 log units (2 lines). Anisometropia was ≥1.00 DS difference in spherical equivalent refraction (SER). Participants with manifest or intermittent strabismus were excluded, however primary microtropia was accepted.
- **Anisometropic Control group** (anisometropia without amblyopia): had BCVA of ≥0.10 logMAR in each eye and less than two lines difference between the eyes. They may have previously undergone amblyopia treatment and achieved best-corrected visual acuity of 0.10 logMAR or better in the amblyopic eye. Anisometropia was ≥1.00 dioptre in SER. All participants in this group had no manifest strabismus and normal binocular vision, as determined by normal horizontal and vertical fusional vergence amplitudes and stereoacuity of 100 secs of arc or better on the Randot Preschool Stereoacuity Test (Stereo Optical.co.inc).
- **Isometric Control group** (no anisometropia or amblyopia): BCVA ≤ 0.10 logMAR in each eye, no history of amblyopia or other binocular vision disorders, no manifest strabismus, and stereoacuity of 100 secs of arc or better on the Randot Preschool Stereoacuity Test
- Participants in all groups had less than –6.00 DS of myopia and less than +8.00 DS of hyperopia (SER). Astigmatism difference between eyes in any meridian was 3.00 DC or less.

### Study procedure

All participants completed a full clinical assessment, including detailed ocular history, distance best corrected visual acuities measured using the highly standardised E-ETDRS protocol on the Electronic Visual Acuity (EVA) Tester (Beck et al. 2003), cover test, ocular motility, convergence, Bagolini striated glasses at 1/3 metre and 6 metres, and the Randot preschool stereoacuity test (Stereo Optical Co. Inc, Chicago, IL, USA) at 40 cm. Objective visuoscopy and the four-dioptre reflex test were used to assess fixation. Ocular biometry was measured using the LenStar LS 900, retinoscopy (non-cycloplegic) and subjective refraction were completed. All tests were conducted by the same examiner (experienced orthoptist) to ensure consistency, with retinoscopy and subjective refractions verified by an optometrist. If participants were not currently wearing the correct prescription, then all tests were performed with the full subjective refraction in trial frames. Otherwise, the participant’s habitual glasses or contact lenses were used during testing. For amblyopic participants, the non-amblyopic fellow eye was deemed to be dominant. For non-amblyopic participants, eye dominance was determined using hole-in-the-hand test.

Subjective aniseikonia was measured using the following four methods:

- **The Aniseikonia Inspector Version 3 (AI3)** is a computer-based clinical test requiring the direct comparison of two rectangles viewed through red-green anaglyphic glasses. The glasses were worn with the red filter over the right eye for all participants. Testing was conducted at a viewing distance of 45cm. The standard ‘Screen’ procedure was used to measure aniseikonia for targets of 4 and 8 degree field angles. Each measurement consisted of 12 presentations of varying amounts of object size difference, where the participant identified which of the two targets appeared larger using the keyboard. The Aniseikonia Inspector performs a small fixation disparity test before aniseikonia measurement is taken. Two targets are presented and moved relative to each other on the screen correcting small amounts of horizontal and vertical fixation disparities. The participants were then instructed to notify the examiner if the two images moved out of alignment during the test. The test was performed in a dimmed room, and the participant was instructed to keep their head as still as possible throughout testing. The participant was observed to ensure they maintained head position and were encouraged to fix centrally throughout testing.
- **The New Aniseikonia Test booklet (NAT)** (Good-Lite Company, Tokyo, Japan) contains 24 pairs of semi-circle targets presented in 1% magnification increments from 0 to 24%. These are viewed through red-green anaglyphic glasses with the red filter over the right eye. The participant viewed the booklet at 40cm and was asked to find the pair of semi-circles that appeared most equal in size. Each set of semi-circles had a number that indicated the percentage of aniseikonia.
- **Contrast-balanced Aniseikonia Test (CAT)** is a novel psychophysical procedure which allowed participants to make manual adjustments before testing to a) align dichoptic images to compensate for any phorias and b) equalise perceived contrast of dichoptic images to compensate for any suppression. The grayscale semi-circle targets were viewed through 3D glasses at a distance of 45cm, and a 30-trial psi-marginal adaptive staircase was employed (Kontsevich and Tyler 1999; Prins 2013) to determine the threshold of equal perceived image size between the two eyes. The size of objects shown to each eye at this threshold of subjective equality is used to calculate the amount of perceived aniseikonia.
- **The Robertson Technique (RT)** is a modified penlight and Maddox rod technique that measures spectacle-induced aniseikonia via neutralisation of the induced vertical anisophoria. This test differs from the other three as it is a measure of dynamic aniseikonia, not static aniseikonia. A Maddox rod lens is placed over the dominant eye, and the participant viewed a pen torch at one metre with both eyes. The horizontal line image seen by the dominant eye would appear to overlap the pen torch seen by the non-dominant eye if the participant viewed through the optical centres of the lenses. The participant is instructed to hold their head still in this position and the light is moved up or down while the participant follows using eye movements only. If dynamic aniseikonia is present, the light and the line will move apart, with the larger image seen at the more peripheral position. Prisms are then used to measure the amount of vertical anisophoria for specific positions of gaze above and below the optical centre direction. These prism measurements are then used to calculate the amount of dynamic aniseikonia (South et al. 2019). From this anisophoria, the static aniseikonia can be inferred (Remole 1989a; Remole 1989b; Remole A 1996).

All participants wore their full refractive correction where required with the appropriate near addition (if required). Participants requiring trial frame correction were given a minimum of 15 minutes to adapt to the lenses prior to attempting the aniseikonia tests. The order of aniseikonia tests for each participant were determined using a computer-generated random order sequence.

### Statistical Analyses

Results from successfully completed subjective aniseikonia tests were converted to the same units for comparison.

The aniseikonia value was calculated as below:

\frac{\begin{array}{l}
Perceived\ size\ in\ nondominant\ or\ amblyopic\\
eye - Perceived\ size\ in\ dominant\ or\ fellow\ eye
\end{array}}{{Perceived\ size\ in\ dominant\ or\ fellow\ eye}} \times 100\%

The amount of anisometropia for each participant was calculated as ‘signed anisometropia’ based on their refractive error, using the formula ***SE***_*NdE*_–***SE***_*DE*_ (*SE*_*NdE*_ *= Spherical Equivalent Non dominant Eye, SE*_*DE*_ *= Spherical Equivalent Dominant Eye*). We preserved the signed information in this calculation instead of using the clinical convention of the absolute amount of anisometropia, as anisometropic spectacle correction is a contributor to the total amount of aniseikonia, and thus whether the non-dominant eye was wearing a more plus or more minus lens than the dominant eye is important for analyses. Direct values of dynamic aniseikonia calculated from the Robertson Technique were used for comparison to the other static aniseikonia tests.

The association between the four different aniseikonia tests was evaluated using Bland Altman analysis in GraphPad Prism 8.2.1. No literature was available to define acceptable limits of agreement for aniseikonia tests. Pearson correlation coefficient analysis was conducted using SPSS version 25 (IBM). The association between the amount of aniseikonia and the amount of signed anisometropia was evaluated using Pearson correlation coefficient. A p-value of <0.05 was used as the threshold for statistical significance for all tests. No adjustments were made for multiple comparisons.

## Results

Participants included (M = 4, F = 15, age range = 15–52) in the study are summarised in Table 1. Five participants habitually wore glasses and three habitually wore contact lenses, and all had prescriptions less than six months old. Nine participants did not routinely wear correction, requiring trial lenses during testing. Interestingly, the Anisometropic Amblyopia group were the least likely to wear habitual correction, with only two out of seven wearing up-to-date refractive correction. In the Anisometropic Amblyopia group, the average (SD) amount of signed anisometropia was 4.07D (1.54), with an average (SD) of 3.12% (2.96) of aniseikonia and average (SD) acuity of 0.40 (0.20) logMAR in the amblyopic eye. Six out seven of these participants had previously undergone occlusion therapy. In the Anisometropic Control group, the average (SD) amount of signed anisometropia was 0.40D (2.84), with an average (SD) of –0.06% (–0.66) of aniseikonia and –0.03 (0.12) logMAR acuity in the non-dominant eye. Only one participant had previously had occlusion therapy. The Isometropic Control group had an average (SD) of 0.30D (0.32) of signed anisometropia, an average (SD) of 0.17% (0.72) of aniseikonia and an average (SD) acuity of –0.05 (0.10) logMAR units.

**Table 1:** Participant characteristics and aniseikonia test results.

Anisometropic Amblyopia Group														

Study Group	Participant Age (Y)	LogMAR Acuity		Cover Test	Bagolini Striated Glasses	Stereopsis (secs/arc)	Signed Anisometropia (D)*	SE Refractive Error		Method of refractive correction	RT (%)	AI3 (%)	NAT (%)	CAT (%)

		RVA	LVA											

AA03	52	0.50	0.00	2 xp	BSV	400	4.88	5.88	1	Contact lenses with glasses for reading add	–0.60	–1.48	1.01	0.42
AA04	18	–0.10	0.30	Micro	Central Suppression	Nil	5.38	–0.5	4.88	Trial frames	–	–	10.00	3.52
AA05	15	–0.10	0.70	Micro	L Suppression	Nil	5.75	–0.25	5.5	Trial frames	1.79	3.50	3.00	0.05
AA10	23	0.20	–0.10	6 xp	BSV	600	4.75	2.5	–2.25	Trial frames	6.82	10.50	6.38	7.05
AA16	22	–0.10	0.60	Micro	Central Suppression	200	3.75	0.5	4.25	Trial frames	1.82	6.00	2.00	1.76
AA17	20	–0.20	0.30	Ortho	BSV	800	2.00	0.5	2.5	Trial frames	4.33	–1.50	0.00	2.39
AA19	22	–0.10	0.20	Ortho	BSV (with int supp @times)	100	2.00	0	2	Habitual Glasses	–0.50	1.50	2.00	2.06
										**Mean**	**2.28**	**3.09**	**3.49**	**2.33**
										**Std Dev**	**2.87**	**4.65**	**3.50**	**2.41**
**Anisometropic Control Group**														

**Study Group**	**Participant Age (Y)**	**LogMAR Acuity**		**Cover Test**	**Bagolini Striated Glasses**	**Stereopsis (secs/arc)**	Signed Anisometropia (D)*	**SE Refractive Error**		**Method of refractive correction**	**RT (%)**	**AI3 (%)**	**NAT (%)**	**CAT (%)**

		**RVA**	**LVA**											

AC02	28	–0.10	0.10	Ortho	BSV	100	3.00	2.13	5.13	Trial frames	4.53	1.52	0.00	–1.13
AC11	22	–0.20	–0.20	4 xp	BSV	40	3.25	–3.25	–0.75	Contact lenses	0.32	1.52	–3.00	–0.39
AC13	28	–0.10	–0.10	4 xp	BSV	40	–1.50	–0.38	–1.88	Habitual Glasses	1.75	0.00	–1.00	–3.29
AC14	28	0.00	0.10	1 xp	BSV	40	–3.25	–3.38	–6.63	Trial frames	–0.23	–1.50	0.00	0.84
AC18	46	–0.10	0.00	12 xp	BSV	40	–1.63	–0.38	–2	Trial frames	0.63	–1.70	1.00	–0.65
AC23	21	–0.10	–0.10	Ortho	BSV	40	2.50	–2.5	0	Habitual Glasses	1.99	–1.50	–1.00	–0.09
										**Mean**	**1.58**	**0.31**	**–0.74**	**–0.93**
										**Std Dev**	**1.75**	**1.62**	**1.41**	**1.35**
**Isometropic Control Group**														

**Study Group**	**Participant Age (Y)**	**LogMAR Acuity**		**Cover Test**	**Bagolini Striated Glasses**	**Stereopsis (secs/arc)**	Signed Anisometropia (D)*	**SE Refractive Error**		**Method of refractive correction**	**RT (%)**	**AI3 (%)**	**NAT (%)**	**CAT (%)**

		**RVA**	**LVA**											

IC01	31	–0.10	–0.10	1 xp	BSV	40	0.63	–5.38	–4.75	Habitual Glasses	–0.51	–1.50	–1.00	–0.79
IC07	38	0.10	–0.10	Ortho	BSV	40	0.25	0	–0.25	No ref correction	0.01	0.50	0.00	–0.32
IC08	23	0.10	–0.10	Ortho	BSV	40	0.13	–0.25	–0.38	No ref correction	0.12	2.56	1.01	0.14
IC15	28	–0.10	–0.10	Ortho	BSV	40	0.00	–0.5	–0.5	Trial frames	0.00	–0.50	0.00	0.09
IC20	19	–0.20	–0.20	1 xp	BSV	40	0.00	–2.38	–2.38	Habitual Glasses	0.00	0.50	1.00	–0.92
IC22	21	0.00	0.00	ortho	BSV	60	0.75	–0.75	–1.5	Contact Lenses	0.00	–0.50	1.01	3.28
										**Mean**	**–0.06**	**0.18**	**0.34**	**0.25**
										**Std Dev**	**0.22**	**1.39**	**0.82**	**1.55**

* Anisometropia was calculated to match the signed percentage of aniseikonia using the following formula: ***SENdE–SEDE*** (*SENdE* = Spherical Equivalent Non dominant Eye, *SEDE* = Spherical Equivalent Dominant Eye). AA = Anisometropia Amblyopia, AC = Anisometropic Control, IC = Isometropic Control.

Eighteen out of 19 participants were able to complete all 4 subjective aniseikonia tests. Only one participant from the Anisometropic Amblyopia group (AA04) was unable to perform two of the tests (RT and AI3) due to a decompensated phoria and loss of fusion during the tests.

**Figure 1:**
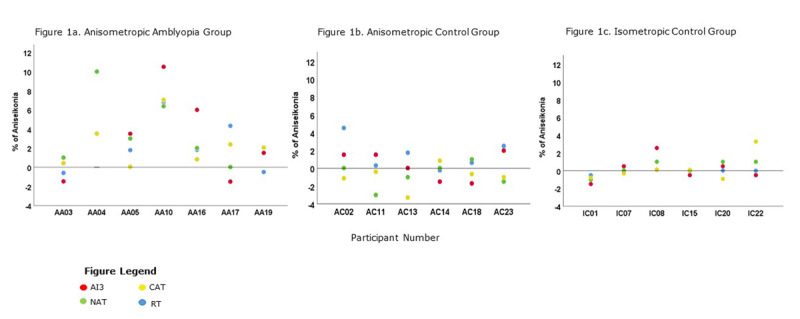
**(a–c) Inter-test Reliability in 3 Study Groups.** Showing greatest amount of aniseikonia in the Anisometropic Amblyopia Group.

The Anisometropic Amblyopia Group (Figure 1a) generally demonstrated the greatest amount of aniseikonia (range –1.50% to +10.50%) followed by Anisometropia Control group (Figure 1b) (range 3.30 to +4.50%) and Isometropic Control group (Figure 1c) (range –1.50 to +3.28%). This is further described for each of the aniseikonia tests in Table 2.

**Table 2:** Range of aniseikonia per test for the 3 study groups.

	AI3	NAT	CAT	RT

**Anisometropia amblyopia**	–1.50% to +10.50%	+0.02% to +10.00%	+0.05% to +7.00%	–0.60% to 6.82%
**Anisometropic Control**	–1.70% to +1.99%	–3.00% to +1.00 %	–3.29% to +0.84%	–0.23% to 4.53%
**Isometropic Control**	–1.50% to +2.56%	–1.00% to +1.00%	–0.92% to 3.28%	–0.51 to 0.1%

**Figure 2:**
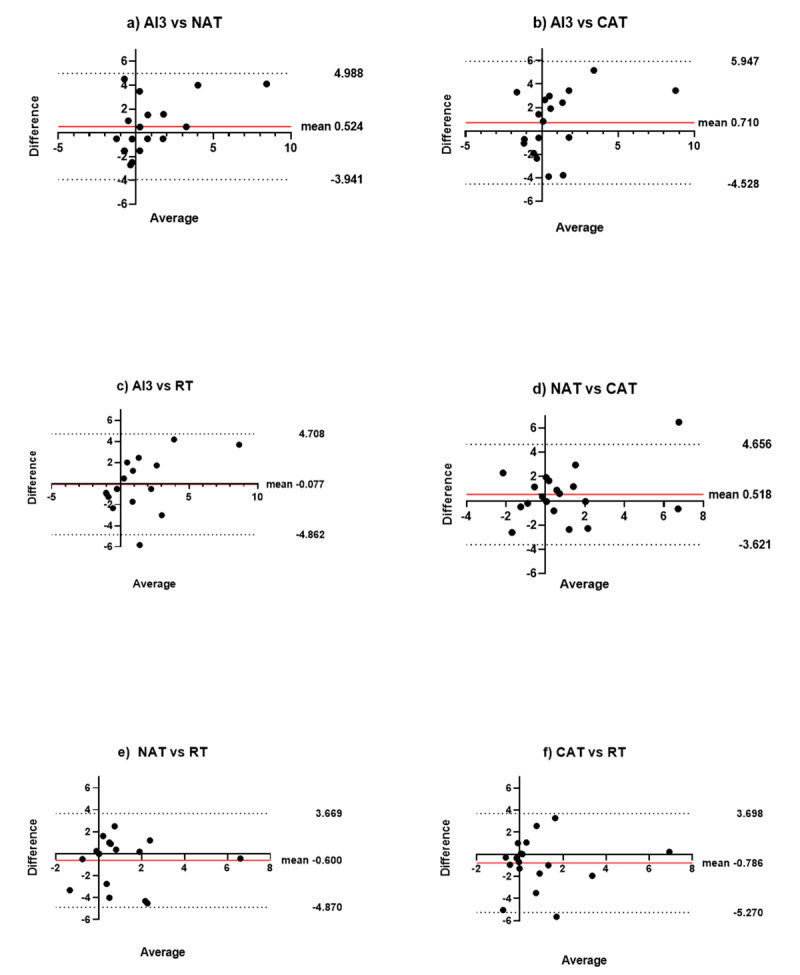
**(a–f)** Bland-Altman plots for repeated measurements of the four aniseikonia tests. The central solid red line shows the mean difference and the upper and lower broken lines show the 95% limits of agreement.

Bland Altman analysis (Figure 2) demonstrated a low level of bias between methods. However, the 95% limits of agreement showed variability, which was greater in the anisometropic amblyopia group.

**Figure 3:**
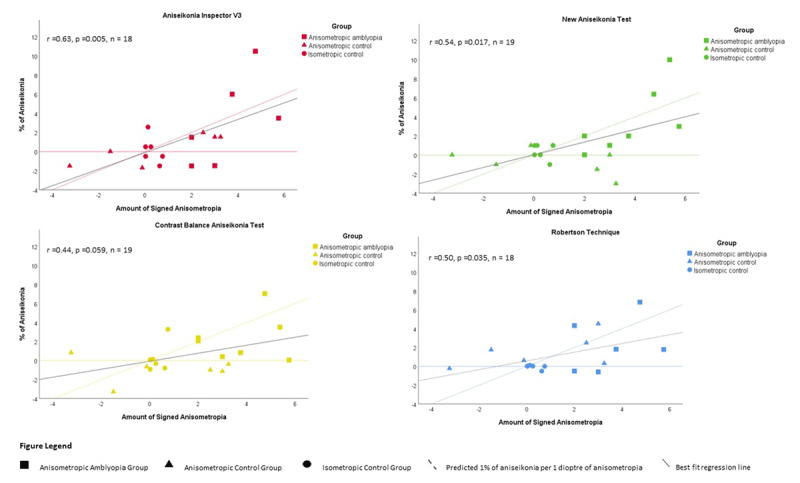
Amount of Aniseikonia versus signed anisometropia.

A significant trend of increasing subjective aniseikonia with increasing amounts of signed anisometropia was observed across all four tests (Figure 3). Three out of the four tests showed significant correlation with the signed anisometropia (AI3 r = 0.63 p = 0.005, NAT r = 0.54 p = 0.017 and RT r = 0.50 p = 0.035). However, the fitted trendlines are all flatter than the ‘1% per Dioptre’ rule of thumb.

## Discussion

Our results show that subjective aniseikonia can be successfully measured in anisometropic amblyopia using both clinical and psychophysical dichoptic methods in our adult cohort. As expected, subjective aniseikonia was correlated with anisometropia and was highest on average in the anisometropic amblyopia group and lowest in the isometropic control group. Our results also demonstrate that the increase in subjective aniseikonia with increase in signed anisometropia does not support the 1% per Dioptre clinical rule of thumb, suggesting that clinical approximations are likely to be inaccurate in anisometropic patients. Actual measurements of subjective aniseikonia should be attempted in patients with anisometropia to provide more precise information for clinical decisions. The three static aniseikonia tests showed good correlation with low levels of bias; however, there were clinically significant levels of variability demonstrated between the tests. This variability was greater in those with anisometropic amblyopia as expected due to difficulty in size judgements associated with worse visual acuity in the amblyopic eye. No test has superseded the Eikonometer, which was the previous gold standard; however, this test is no longer in production and no longer commercially available (Rutstein et al. 2006). The variability found between the tests suggests future studies should aim to provide a gold standard test and to establish clinically acceptable limits of agreement and test-retest variability between aniseikonia tests.

All participants except one were able to perform all four aniseikonia tests. This is the first study demonstrating that aniseikonia tests can be reliably performed in adults with anisometropic amblyopia. Previous studies have excluded participants who did not demonstrate simultaneous perception or stereopsis (Antona et al. 2007; Awaya S 1982; Kehler et al. 2014; Lubkin et al. 1999) as aniseikonia was assumed to be difficult to measure in amblyopia due to suppression. While Lubkin et al. (1999) investigated the relationships between aniseikonia, anisometropia, strabismus, and amblyopia, their participants had very low levels of suppression as they were required to have stereopsis of 100 secs of arc or better to perform Space Eikonometry. Our study successfully measured subjective aniseikonia even in the anisometropic amblyopia group, which included participants with residual amblyopia, reduced or nil stereoacuity, and demonstrable suppression on Bagolini lenses.

Chronic suppression in anisometropic amblyopia develops due to the diminished image clarity and contrast in one eye during the early critical period of visual development (Levi et al. 2011). Recent binocular theories of amblyopic visual deficits suggest that binocular visual function is suppressed or inactive under normal viewing conditions and not permanently lost. Our results support this theory, as our participants with anisometropic amblyopia were able to complete direct comparison tests, which require binocular simultaneous perception. It is possible that they were able to overcome suppression simply through red/green anaglyphic dissociation or by viewing contrast-balanced dichoptic stimuli. Suppression in anisometropic amblyopia is also spatial frequency dependent, with more unbalanced suppression at higher spatial frequencies (Kwon et al. 2015; Movshon et al. 1987). The tests we used all had relatively large, solid shapes and minimal high-spatial frequency textures, which should stimulate less suppression than finely detailed gratings or small targets. The dichoptic shapes also did not overlap in visual space, preventing binocular rivalry, and were framed by binocular stimuli to encourage peripheral fusion. All of these factors allow for the measurement of aniseikonia even in the presence of amblyopic suppression.

The Robertson Technique uses simple equipment that is already found in most orthoptic clinics to measure dynamic aniseikonia (Remole 1989a; Remole, 1989b), and a static percentage difference can be derived from this measurement (Remole 1989b). Remole (1989a) suggested correcting two-thirds of measured dynamic aniseikonia should provide overall symptomatic relief. However, looking at our results, correcting two-thirds of the dynamic amount would result in under or overcorrection of the aniseikonia for some participants. Contact lenses have been shown to be effective in reducing symptoms of both static and dynamic aniseikonia (Rose and Levinson 1972; Winn et al. 1988). Contact lenses sit closer to the entrance pupil than spectacles reducing the optically induced magnification effect caused by lens power. Contact lenses also remain centred on the cornea during eye movements and therefore dynamic aniseikonia is not induced. However, they are not always suitable for all patients, such as elderly patients or young children. The range of refractive correction used in this study including contact lenses, habitual glasses, and trial frames could account for the variability in the range of aniseikonia measured in the three study groups (see Table 2). The majority of participants in the anisometropic amblyopia group had trial frame corrections as they were wearing balance lens prescriptions or were not routinely using any refractive correction. Clinically, deliberate under correction using reduced-power balance lenses are often prescribed to adult patients with large amounts of anisometropia to reduce the risk of aniseikonia symptoms. In most cases, the actual amount of aniseikonia perceived by the patient is not assessed. Deliberate under correction of anisometropia or not correcting anisometropia without assessing aniseikonia deprives patients of binocular vision.

One participant with anisometropic amblyopia was unable to complete the AI3 and RT due to decompensation of a horizontal and vertical phoria during testing and intermittent central suppression. The AI3 allowed correction of small horizontal and vertical fixation disparities up to 4 secs of arc, but our participant’s vertical disparity was beyond this limit. Vertical prisms placed in trial frames were used in an attempt aid fusion during both the AI3 and RT tests, but poor motor fusion resulted in intermittent diplopia, triggering suppression and making it difficult to perceive and maintain alignment of the targets. Interestingly, the participant was able to appreciate some image size differences between eyes during moments without suppression and was able to perform the NAT. It is likely this participant was not maintaining central fixation and therefore not truly performing a size discrimination task (Garcia-Perez and Peli 2015). Alternating suppression may have allowed for the image sizes to be perceived uniocularly and a comparison made. The opposing half circle targets and colour contrast of the NAT may have further helped identify which eye was seeing which image while uniocular comparisons were made. The CAT allowed a larger adjustment of vertical and horizontal alignment to compensate adequately for this participant’s phoria, and contrast adjustment to overcome suppression, allowing a measurement of subjective static aniseikonia to be obtained. This participant’s example illustrates the difficulty in performing dichoptic tests in patients with abnormal motor and sensory binocularity. An optimal aniseikonia test needs to be able to overcome issues such as loss of fusion and suppression.

The two more well-known clinical aniseikonia tests, NAT and the AI3, showed good correlation between tests r = 0.679 p = 0.002 and a low level of bias. The newly developed CAT also showed significant correlation with both these tests (NAT r = 0.689 p = 0.001, AI3 r = 0.536 p = 0.022), and the Robertson technique shows good correlation with the AI3 (Table 3, mean -0.08 r = 0.618 p = 0.006), which provides a good clinical test alternative using equipment that is already found in most orthoptic clinics compared to expensive software purchases. These tests all measure static aniseikonia using direct comparison of perceived image size under dichoptic conditions, and therefore similar results are expected. Overall, the AI3 and the NAT static aniseikonia tests appear to be useful in anisometropic amblyopia, and limitations to direct comparison methods may be addressed by further refinement of digital or paper-based methods (such as the CAT test).

**Table 3:** Results of Bland-Altman analysis for repeated measurements and Pearson correlation values of the four aniseikonia tests.

Bland Altman results for repeated measures of the four aniseikonia tests			Pearson Correlation Coefficient values	

Test	Mean Difference (%)	95% limits of agreement (%)	R -values	P-value

AI3 vs. NAT	0.52	–3.94 to 5.00	0.679	0.002
AI3 vs. CAT	0.71	–4.53 to 5.95	0.536	0.022
AI3 vs. RT	–0.08	–4.86 to 4.71	0.618	0.006
NAT vs. CAT	0.52	–3.62 to 4.66	0.689	0.001
NAT vs. RT	–0.60	–4.87 to 3.67	0.432	0.073
CAT vs. RT	–0.79	–5.27 to 3.70	0.434	0.072

The study shows a significant trend of increasing subjective aniseikonia with increasing amounts of anisometropia but does not support the 1% per dioptre clinical rule, which was also reported by Lubkin et al. (1999). This suggests other factors, such as cortical and retinal adaptations, may be contributing to the final perceived amount of aniseikonia, and empirical calculations alone do not provide an accurate solution for the management of aniseikonia. We acknowledge the small sample size in this study does not allow for an accurate calculation of average percentage of aniseikonia per dioptre of anisometropia, and a larger sample size would be required for a true estimation. Recruitment of participants within the three groups with the specific criteria was challenging; however, the sample size in this study is similar to other recent studies in this area (Atchison et al., 2020; Primiano Junior et al. 2019). A larger population level study is an area that requires further research and would increase the power and reduce the margins of error. Lubkin and Linksz (1977) studied the interrelationships among aniseikonia, anisometropia, strabismus, and amblyopia and noted a 4.4-fold increased risk of aniseikonia in anisometropia but did not look at the degree of aniseikonia in relation to the amount of anisometropia. As far as we know, the quantitative relationship between aniseikonia and anisometropia in those with anisometropic amblyopia has not been studied. However, despite being small in scale, our study suggests that aniseikonia is likely to be common in those with anisometropic amblyopia. Much larger cohorts would be needed to examine the true prevalence. It is promising that simple clinical tests can be used to measure subjective aniseikonia in this patient population.

## Conclusion

Aniseikonia is likely to be present in patients with anisometropia due to the inherent anatomical causes of anisometropia and spectacle corrections used for treatment. We have shown that aniseikonia occurs in patients with anisometropic amblyopia and that subjectively perceived aniseikonia can be reliably measured despite amblyopia and suppression. The greater amounts of aniseikonia found in the anisometropic amblyopia group is in line with the hypothesis that aniseikonia may contribute to suppression and may limit binocular visual recovery in anisometropic amblyopia. It is possible that correcting aniseikonia simultaneously with anisometropia at first diagnosis will reduce the need to develop suppression and improve the overall visual outcomes from amblyopia treatments. To investigate this hypothesis, a randomised clinical trial that directly compares visual outcomes from aniseikonia correction versus standard spectacle correction for anisometropic amblyopia, titled Measuring Aniseikonia: investiGating Neuroplasticity and Image Factors in amblYopia (MAGNIFY) study (ACTRN12620000061932), is currently underway. Further investigations into whether providing aniseikonia correction in older children/adults with anisometropic amblyopia could improve spectacle compliance and visual function would also contribute to the understanding of the role of aniseikonia in anisometropic amblyopia.

## Additional File

The additional file for this article can be found as follows:

10.22599/bioj.154.s1 Supplementary material. Participant Raw Refraction Data.
