# Supplementary material for: Clinical Aniseikonia in Anisometropia and Amblyopia
Source: Br Ir Orthopt J. 2020 Nov 20;16(1):44–54. doi: 10.22599/bioj.154 (PMC8269785; doi:10.22599/bioj.154)
Supplement: Supplementary material. — Participant Raw Refraction Data. [file bioj-16-1-154-s1.pdf]

Supplementary material

Participant Raw Refraction Data

Anisometropic Amblyopia Group		Sphere	Cylinder	Axis	SE
AA03	R	+6.00	-0.25	105	5.88
	L	+1.00	0	180	1.00
AA04	R	-0.25	-0.50	180	-0.50
	L	+5.50	-1.25	5	4.88
AA05	R	-0.25	0	180	-0.25
	L	+6.50	-2.00	180	5.50
AA10	R	+2.50	0	180	2.50
	L	-2.25	0	180	-2.25
AA16	R	+0.50	0	180	0.50
	L	+4.25	0	180	4.25
AA17	R	+0.50	0	180	0.5
	L	+2.75	-0.50	45	2.50
AA19	R	-0.25	0	180	-0.25
	L	+2.00	0	180	2.00
Anisometropic Control Group		Sphere	Cylinder	Axis	SE
AC02	R	+2.25	-0.25	40	2.13
	L	+5.50	-0.75	115	5.13
AC11	R	-3.25	0	180	-3.25
	L	-0.75	0	180	-0.75
AC13	R	-0.25	-0.25	90	-0.38
	L	-1.75	-0.25	100	-1.88
AC14	R	-2.75	-1.25	165	-3.38
	L	-5.50	-2.25	165	-6.63
AC18	R	-0.25	-0.25	75	-0.38
	L	-0.75	-2.50	9	-2.00
AC23	R	-2.50	0	180	-2.50
	L	0	0	180	0.00
Isometropic Control Group	L	Sphere	Cylinder	Axis	SE
IC01	R	-5.25	-0.25	105	-5.38
	L	-4.75	0	180	-4.75
IC07	R	0	0	180	0.00
	L	0	0	180	0.00
IC08	R	0	-0.5	95	-0.25
	L	-0.25	-0.25	74	-0.38
IC15	R	-0.50	0	180	-0.50
	L	-0.50	0	180	-0.50
IC20	R	-2.00	-0.75	90	-2.38
	L	-1.75	-0.75	95	-2.13
IC22	R	-1.00	0	180	-1.00
	L	-1.75	0	180	-1.75
